# Pericoronary fat inflammation and Major Adverse Cardiac Events (MACE) in prediabetic patients with acute myocardial infarction: effects of metformin

**DOI:** 10.1186/s12933-019-0931-0

**Published:** 2019-09-30

**Authors:** Celestino Sardu, Nunzia D’Onofrio, Michele Torella, Michele Portoghese, Francesco Loreni, Simone Mureddu, Giuseppe Signoriello, Lucia Scisciola, Michelangela Barbieri, Maria Rosaria Rizzo, Marilena Galdiero, Marisa De Feo, Maria Luisa Balestrieri, Giuseppe Paolisso, Raffaele Marfella

**Affiliations:** 1 Department of Advanced Medical and Surgical Sciences, University of Campania “Luigi Vanvitelli”, Piazza Miraglia, 2, 80138 Naples, Italy; 2 Department of Precision Medicine, University of Campania “Luigi Vanvitelli”, Naples, Italy; 3 Department of Translational Medical Sciences, University of Campania “Luigi Vanvitelli”, Naples, Italy; 4 Department of Cardiac Surgery, Santissima Annunziata Hospital, Sassari, Italy; 5 Department of Mental Health and Public Medicine, Section of Statistic, University of Campania “Luigi Vanvitelli”, Naples, Italy; 6 Department of Experimental Medicine, University of Campania “Luigi Vanvitelli”, Naples, Italy

**Keywords:** Prediabetes, Acute myocardial infarction, Metformin, Pericoronary fat, Inflammation, Adipokines

## Abstract

**Background/objectives:**

Pericoronary adipose tissue inflammation might lead to the development and destabilization of coronary plaques in prediabetic patients. Here, we evaluated inflammation and leptin to adiponectin ratio in pericoronary fat from patients subjected to coronary artery bypass grafting (CABG) for acute myocardial infarction (AMI). Furthermore, we compared the 12-month prognosis of prediabetic patients compared to normoglycemic patients (NG). Finally, the effect of metformin therapy on pericoronary fat inflammation and 12-months prognosis in AMI-prediabetic patients was also evaluated.

**Methods:**

An observational prospective study was conducted on patients with first AMI referred for CABG. Participants were divided in prediabetic and NG-patients. Prediabetic patients were divided in two groups; never-metformin-users and current-metformin-users receiving metformin therapy for almost 6 months before CABG. During the by-pass procedure on epicardial coronary portion, the pericoronary fat was removed from the surrounding stenosis area. The primary endpoints were the assessments of Major-Adverse-Cardiac-Events (MACE) at 12-month follow-up. Moreover, inflammatory tone was evaluated by measuring pericoronary fat levels of tumor necrosis factor-α (TNF-α), sirtuin 6 (SIRT6), and leptin to adiponectin ratio. Finally, inflammatory tone was correlated to the MACE during the 12-months follow-up.

**Results:**

The MACE was 9.1% in all prediabetic patients and 3% in NG-patients. In prediabetic patients, current-metformin-users presented a significantly lower rate of MACE compared to prediabetic patients never-metformin-users. In addition, prediabetic patients showed higher inflammatory tone and leptin to adiponectin ratio in pericoronary fat compared to NG-patients (P < 0.001). Prediabetic never-metformin-users showed higher inflammatory tone and leptin to adiponectin ratio in pericoronary fat compared to current-metformin-users (P < 0.001). Remarkably, inflammatory tone and leptin to adiponectin ratio was significantly related to the MACE during the 12-months follow-up.

**Conclusion:**

Prediabetes increase inflammatory burden in pericoronary adipose tissue. Metformin by reducing inflammatory tone and leptin to adiponectin ratio in pericoronary fat may improve prognosis in prediabetic patients with AMI.

*Trial registration* Clinical Trial NCT03360981, Retrospectively Registered 7 January 2018

## Background

Prediabetes affects more than 38% of people in adult population [1]. Without any intervention, prediabetes often progresses to diabetes mellitus and is associated with increased risk of cardiovascular disease [2]. Although indices of glycemia define clinical criteria for prediabetes, vascular dysfunction results not only from effects of hyperglycemia but also from vascular proinflammatory and metabolic consequences associated to increased adipose tissue in diverse anatomic locations [3]. Adipose tissue is an active endocrine organ secreting multiple metabolically active factors, such as leptin, interleukin-6 (IL-6) and tumor necrosis factor-α (TNF-α) [4]. These factors are involved in metabolic and inflammatory processes and may act in a paracrine or endocrine manner, altering liver, pancreas, skeletal muscle and vascular system metabolism [5]. Dysregulation of these signal molecules is closely related to adipocyte hypertrophy and insulin resistance [6, 7]. In humans, coronary arteries are the most atherosclerosis-prone arteries with abundant of adipose tissue surrounding [3]. Recently, pericoronary fat has been considered as an active component of the blood vessel walls, as it is involved in vascular homeostasis and in atherosclerosis progression [3, 8]. In addition, epicardial adipose tissue in patients with advanced coronary artery disease (CAD) subjected to coronary artery bypass grafting (CABG) releases higher levels of inflammatory cytokines, such as TNF-α than the subcutaneous tissue [3]. Epicardial adipose tissue is also an important source of adiponectin, an anti-inflammatory and anti-atherogenic adipokine [9]. Remarkably, adipocyte-specific SIRT6 knockout mice showed an increased inflammation grade in the adipose tissue along with body weight increase and systemic insulin resistance [10, 11]. In contrast, SIRT6 overexpression resulted in the reduction of the fat mass, lowering LDL cholesterol and triglyceride levels, and improving glucose tolerance [11]. SIRT6 knockout mice showed also an increased expression of inflammatory genes, TNF-α, IL-6, and monocytes chemoattractive protein-1 (MCP-1) in both white and brown adipose tissues [12]. Lastly, SIRT6 is involved in the inflammatory pathways of diabetic atherosclerotic lesions [13]. Thus, is reasonable that inflammatory cytokines and adipokines released from pericoronary fat may be involved in the outcomes of prediabetic patients after cardiovascular events, including coronary artery diseases. To date, the specific molecular mechanisms regulating the inflammatory state of pericoronary fat in prediabetic patients and the possible effect of metformin therapy are poorly investigated. Moreover, there are no study describing the effects of the pre-diabetic milieu on pericoronary fat in patients with acute myocardial infarction (AMI). It is conceivable that pericoronary fat in patients with prediabetes may release inflammatory factors leading to the development and destabilization of atherosclerotic plaques in coronary arteries. In this setting, the aim of this study was to investigate the pro-inflammatory tone in pericoronary fat, by evaluating leptin to adiponectin ratio, TNF-α levels, and SIRT6 protein level [14]. Subsequently, we evaluated the 12-months prognosis after the event by comparing prediabetic to normoglycemic patients subjected to CABG for AMI. Finally, since metformin therapy regulates both leptin and adiponectin levels in plasma and in subcutaneous fat [15–17], here, its effect on leptin to adiponectin ratio and inflammatory tone in pericoronary fat was also evaluated, as well as its correlation with 12-months prognosis in AMI-prediabetic patients.

## Methods

We conducted an observational, multicenter, prospective study. Consecutive patients with first uncomplicated AMI and multivessel coronary artery disease referred for coronary artery bypass graft (CABG), entered prospectively into the database. Criteria for exclusion encompassed concomitant chronic diseases, including kidney, liver, severe uncontrolled hypertension (blood pressure > 200/100 mmHg), routinely consuming more than 3 alcoholic drinks per day, uncontrolled endocrine or metabolic disease known to influence glycemia (i.e., secondary causes of hyperglycemia), congestive heart failure defined by NYHA (New York Heart Association) Class III or IV, ileal bypass, gastric bypass, or other significant intestinal malabsorption, estimated glomerular filtration rate (eGFR) < 30 ml/min/1.73 m^2^ based on the 4-variable MDRD (Modification of Diet in Renal Disease), nephrotic syndrome or other clinically significant renal disease, disorders of the hematologic, digestive, or central nervous systems including cerebrovascular disease and degenerative disease that would limit study evaluation or participation. Because BMI > 35 kg/m^2^ is strongly associated with CABG operative mortality [18], we excluded patients with BMI > 35 kg/m^2^ to avoid bias in the data analyses. Participants were divided in prediabetic patients and normal glucose (NG) patients. Prediabetes was defined by impaired fasting glucose, impaired glucose tolerance and HbA1c value ≥ 5.7% but < 6.5% [19]. Among prediabetic patients, patients who never used metformin were classified as “never-metformin-users.” Prediabetic patients who had already used metformin for at least 6 months before the event and continued throughout the follow up were classified as “current-metformin-users”. Prediabetic patients who had been using metformin for less that 6 months were excluded from the study. Pro-inflammatory tone, defined as TNF-α, reduced SIRT6 levels, and leptin to adiponectin ratio were determined in pericoronary fat. During the by-pass procedure on epicardial coronary portion, the pericoronary fat was removed from the surrounding stenosis portion and from the portions of fat surrounding the segment of coronary identified for the post-stenotic anastomosis, according to the normal technical procedures of the intervention. After removing, the fat was stored in tubes with a RNA-Lather solution. Pericoronary fat tissue (100 mg) was cut into small pieces and homogenized. Fat tissues were lysed and centrifuged for 10 min at 10,000*g* at 4 °C. After centrifugation, each sample were loaded, electrophoresed in polyacrylamide gel, and electroblotted onto a nitrocellulose membrane. Each determination was repeated at least three times. TNF-α, SIRT6, leptin and adiponectin levels were measured in 100 μg of the protein extract from pericoronary fat specimens, using specific TNF-α assays (Cymax human TNF-alpha ELISA, YIF-LF-EK0193, Liestal Switzerland), SIRT6 assay (Mybiosouce MBS2021864 Sirtuin 6 ELISA Kit, San Diego, USA), leptin assay (Human Leptin ELISA Kit ab100581, abcam, Cambridge CB2 0AX UK) and adiponectin assay (Human Adiponectin ELISA Kit ab99968, abcam, Cambridge CB2 0AX UK). The primary endpoints were the assessment of inflammatory tone and Major-Adverse-Cardiac-Events (MACE: all death, cardiovascular death, re-infarction and heart failure) at 12-months follow-up. Heart failure (HF) was defined according to clinical and echocardiographic parameters for HFrEF and HFpEF [20] All patients underwent quarterly clinical evaluation and routine analyses as outpatients for 12-months after the event. The study was conducted at Department of Advanced Medical and Surgical Sciences, Università degli Studi della Campania “Luigi Vanvitelli”, Italy, at Department of Cardio-Thoracic and Respiratory Sciences, Università degli Studi della Campania “Luigi Vanvitelli”, Italy, and at Department of Cardiac Surgery, Santissima Annunziata Hospital, Sassari, Italy. The study was conducted in accordance with the Declaration of Helsinki. The Ethics Committees of all participating institutions approved the protocol (Ethic Committee Università degli Studi della Campania “Luigi Vanvitelli” number: 341). All patients were informed about the study nature, and gave their written informed, and signed consent to participate in the study.

### Statistical analysis

SPSS version 23.0 (IBM statistics) was used for all statistical analyses. Categorical variables were presented as frequencies (percentages), and continuous variables as mean ± SD. The normal distribution of data was evaluated with Kolmogorov Smirnov test and parametric test was used. For comparison among prediabetics and NG-patients, pre-diabetic never-metformin-users and current-metformin-users, a propensity score matching (PSM) was developed from the predicted probabilities of a multivariable logistic regression model predicting mortality, and events from age, sex, hypertension, dyslipidemia, smoking history, family history, baseline no-diabetic therapies, metabolic parameters, and coronary lesions. Pre-diabetic never-metformin-users-users were matched to and current-metformin-users on the basis of PSM. In all matched patients, the balancing property was satisfied. Overall survival and event-free survival are presented using Kaplan–Meier survival curves, and compared using the log-rank test. To investigate the effects of TNFα, SIRT6 levels and leptin to adiponectin ratio on cardiovascular endpoints, we evaluated AMI outcomes at 1-year follow-up stratified by TNFα, SIRT6 and leptin to adiponectin ratio terziles. Regression analysis was performed for estimating the relationships among variables. Sample size was obtained at follow-up. A sample size of 116 patients and 68 events are sufficient to determine a hazard ratio of 0.31 to detect the comparison between group Pre-diabetic and Group NG with a power of 80 percent and a significance level of 5%. A sample size of 360 patients and 28 events are sufficient to determine a hazard ratio of 0.31 to detect the comparison between group PDM ad DC group with a power of 80% and a significance level of 5%. Finally, we analyzed separately the effect of the three glycemic criteria, in accordance with the guideline from ADA [19], on inflammatory tone and leptin to adiponectin ratio, as well as on cardiovascular endpoints. A 2-tailed P value < 0.05 was considered statistically significant.

## Results

**Fig. 1:**
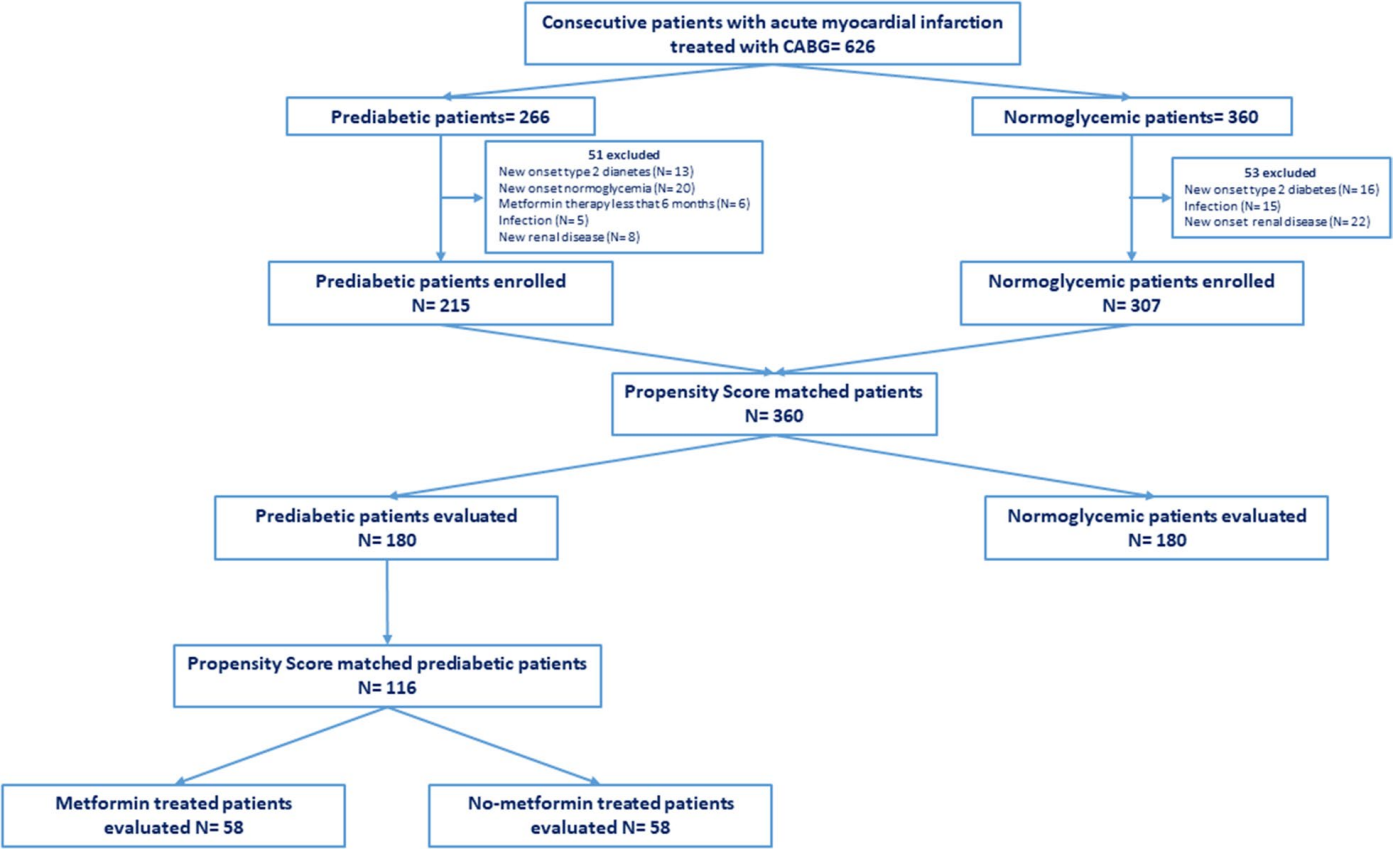
Flow-chart of the study protocol

**Fig. 2:**
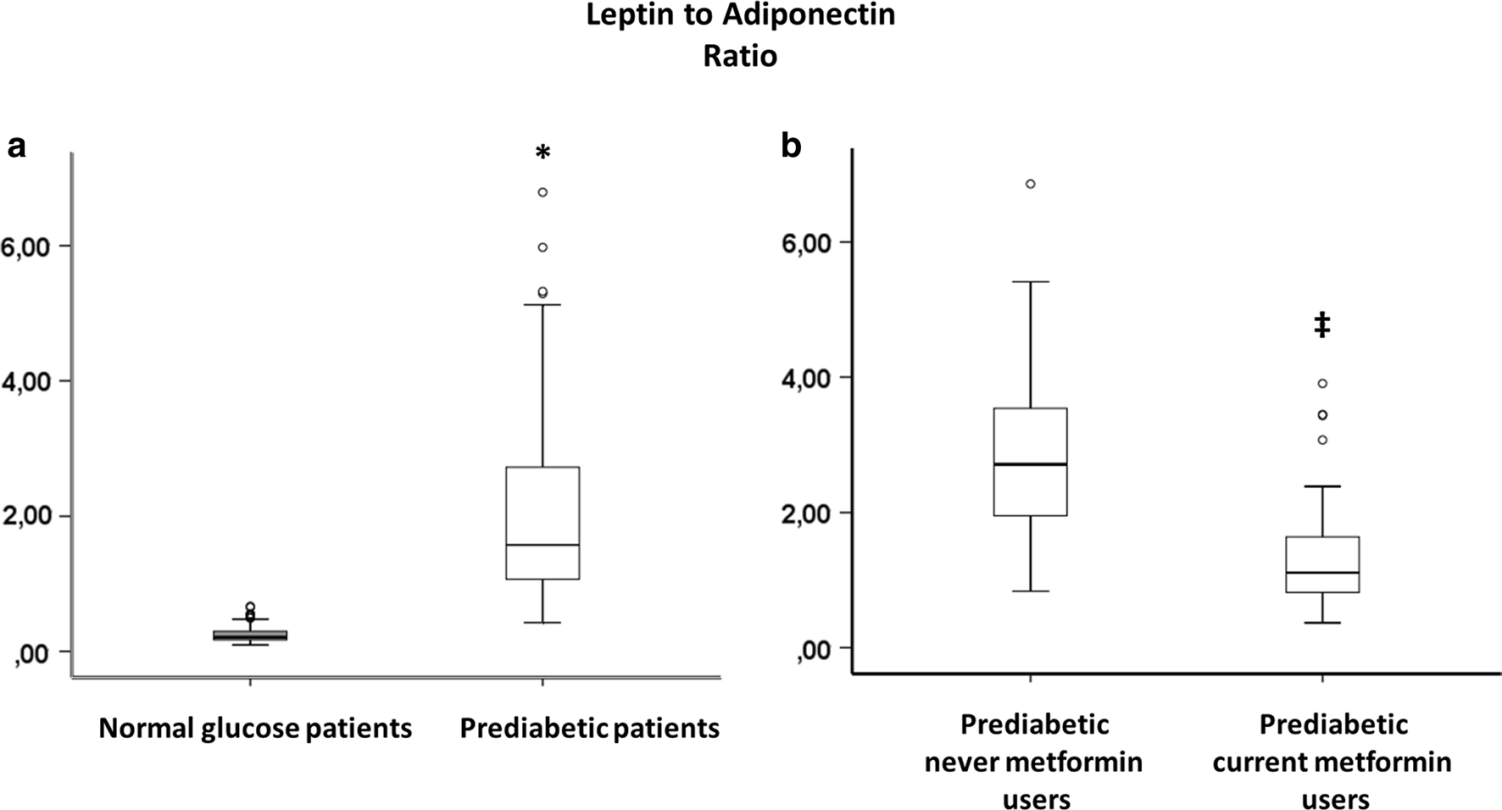
**a** Leptin to adiponectin ratio, in pericoronary fat specimens from 180 normal glucose patients and 180 prediabetic patients matched with propensity score analysis (PSM). (Boxplot, a plot type that displays the median, 25th, and 75th percentiles and range). *P < 0.01 vs. normal glucose patients. **b** Leptin to adiponectin ratio, in pericoronary fat specimens from 58 prediabetic never metformin users, and 58 prediabetic metformin users. ^‡^P < 0.01 vs. never metformin users. Data are mean ± SD

**Fig. 3:**
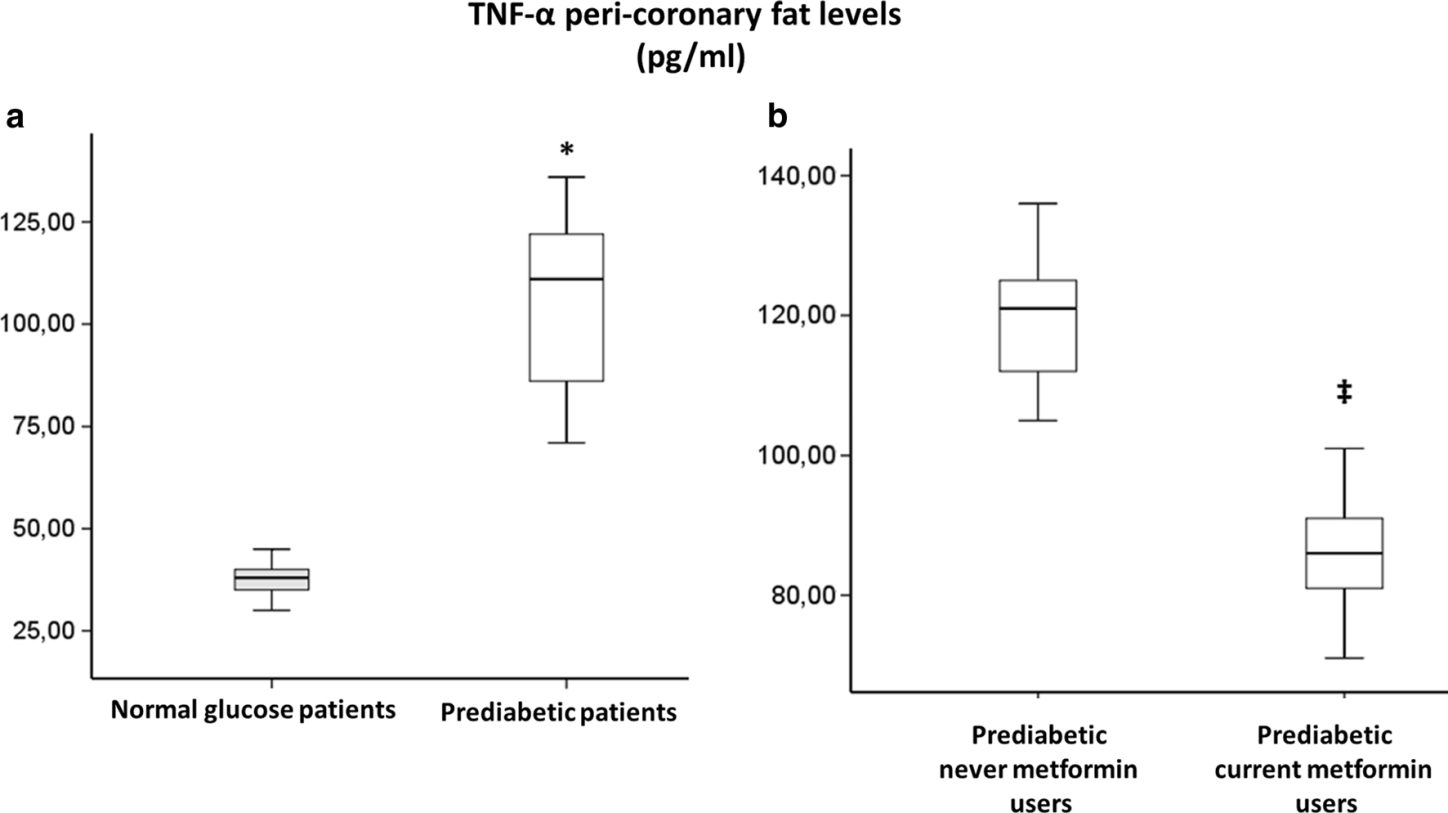
**a** Tumor necrosis factor-α (TNF-α) levels, in pericoronary fat specimens from 180 normal glucose patients and 180 prediabetic patients *P < 0.01 vs. normal glucose patients. **b** TNF-α levels, in pericoronary fat specimens from 58 prediabetic never metformin users and current metformin users. ^‡^P<0.01 vs. never metformin users. Data are mean ± SD

**Fig. 4:**
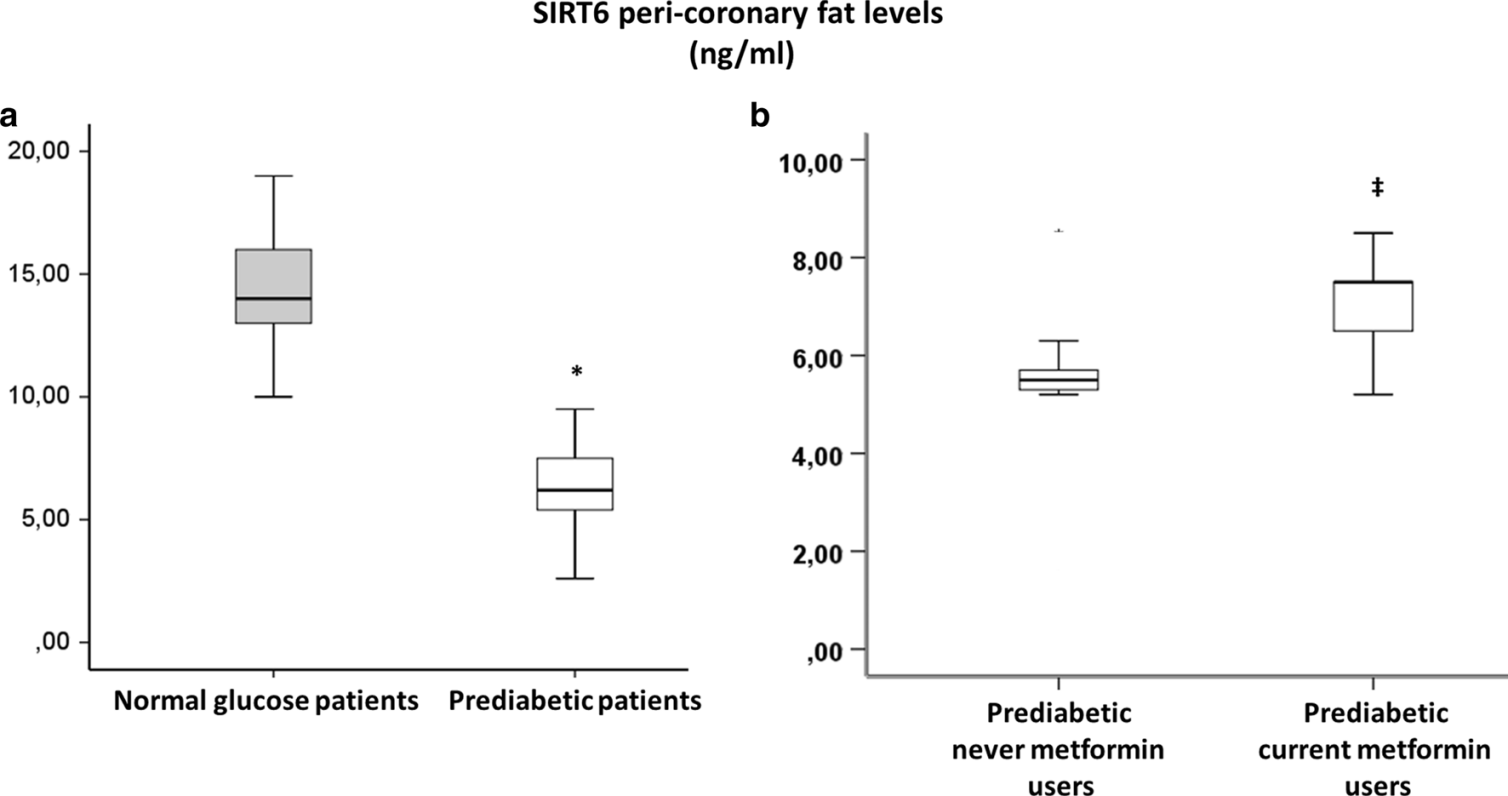
**a** Sirtuin-6 (SIRT6) levels, in pericoronary fat specimens from 180 normal glucose patients and 180 prediabetic patients matched with propensity score analysis (PSM). *P < 0.01 vs. normal glucose patients. **b** SIRT6 levels, in pericoronary fat specimens from 58 prediabetic never metformin users, and 58 prediabetic metformin users. ^‡^P < 0.01 vs never metformin users. Data are mean ± SD

**Fig. 5:**
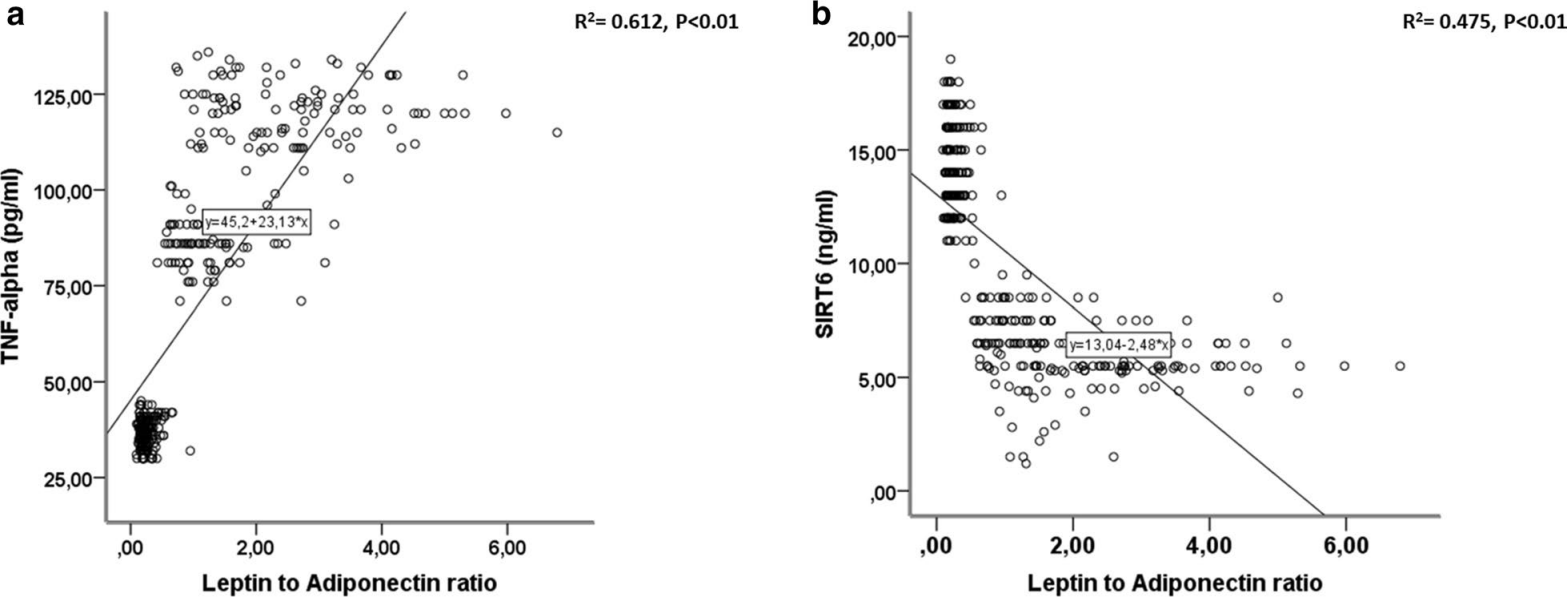
**a** Regression analysis evidences a relationship between pericoronary fat leptin to adiponectin ratio and Tumor necrosis factor-α (TNF-α) levels in the overall study population. This analysis showed that the values of pericoronary TNF-α content (dependent variables) changed when pericoronary fat leptin to adiponectin ratio (independent variable) varied, while the other independent variables are held fixed. **b** Regression analysis evidences a relationship between pericoronary fat leptin to adiponectin ratio and sirtuin 6 (SIRT6) levels in the overall study population. This analysis showed that the values of pericoronary SIRT6 content (dependent variables) changed when fat pericoronary leptin to adiponectin ratio (independent variable) varied, while the other independent variables are held fixed

**Fig. 6:**
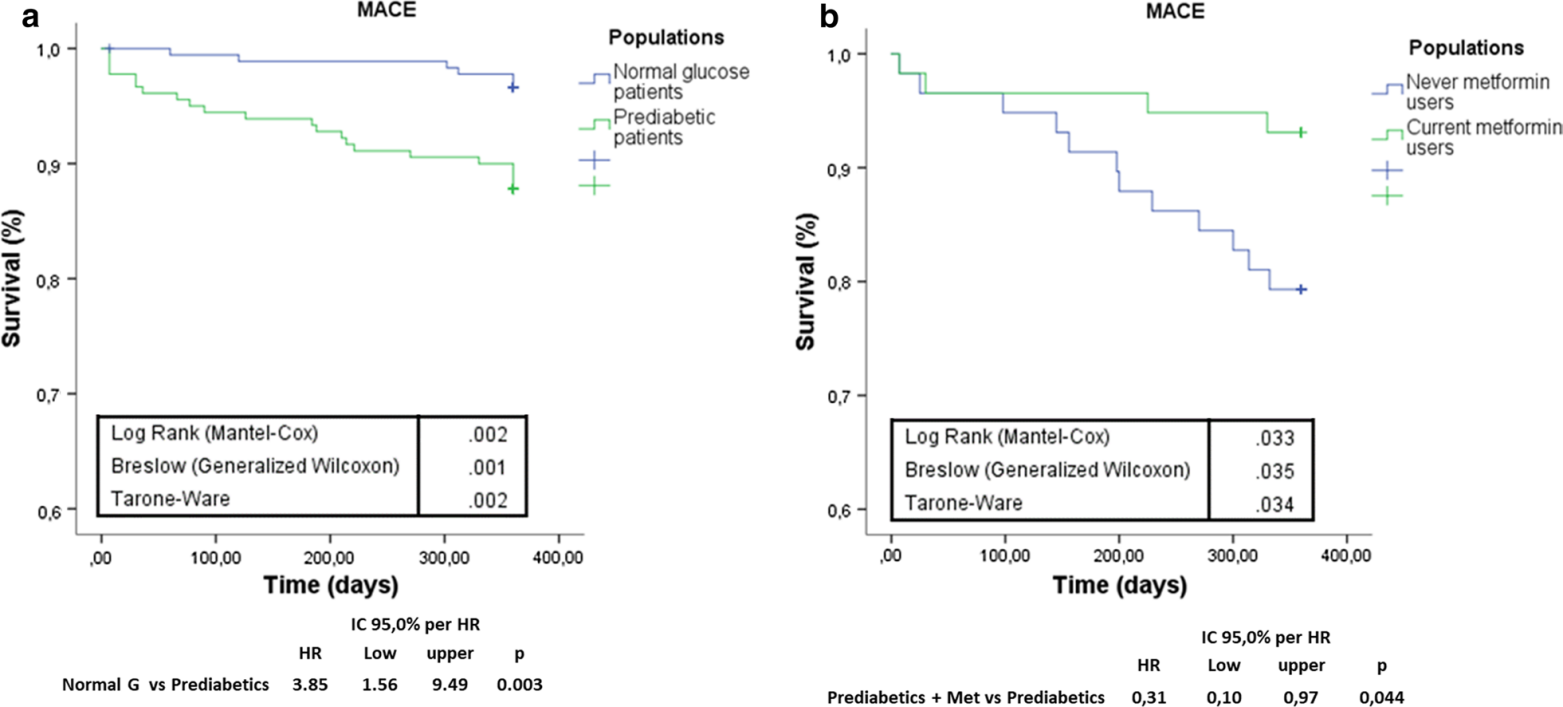
**a** Kaplan–Meier survival curves in PSM normal glucose and prediabetic patients. **b** Kaplan–Meier survival curves in PSM prediabetic never metformin users and prediabetic current metformin users. Overall survival and event-free survival are presented using, and compared using the log-rank test

**Fig. 7:**
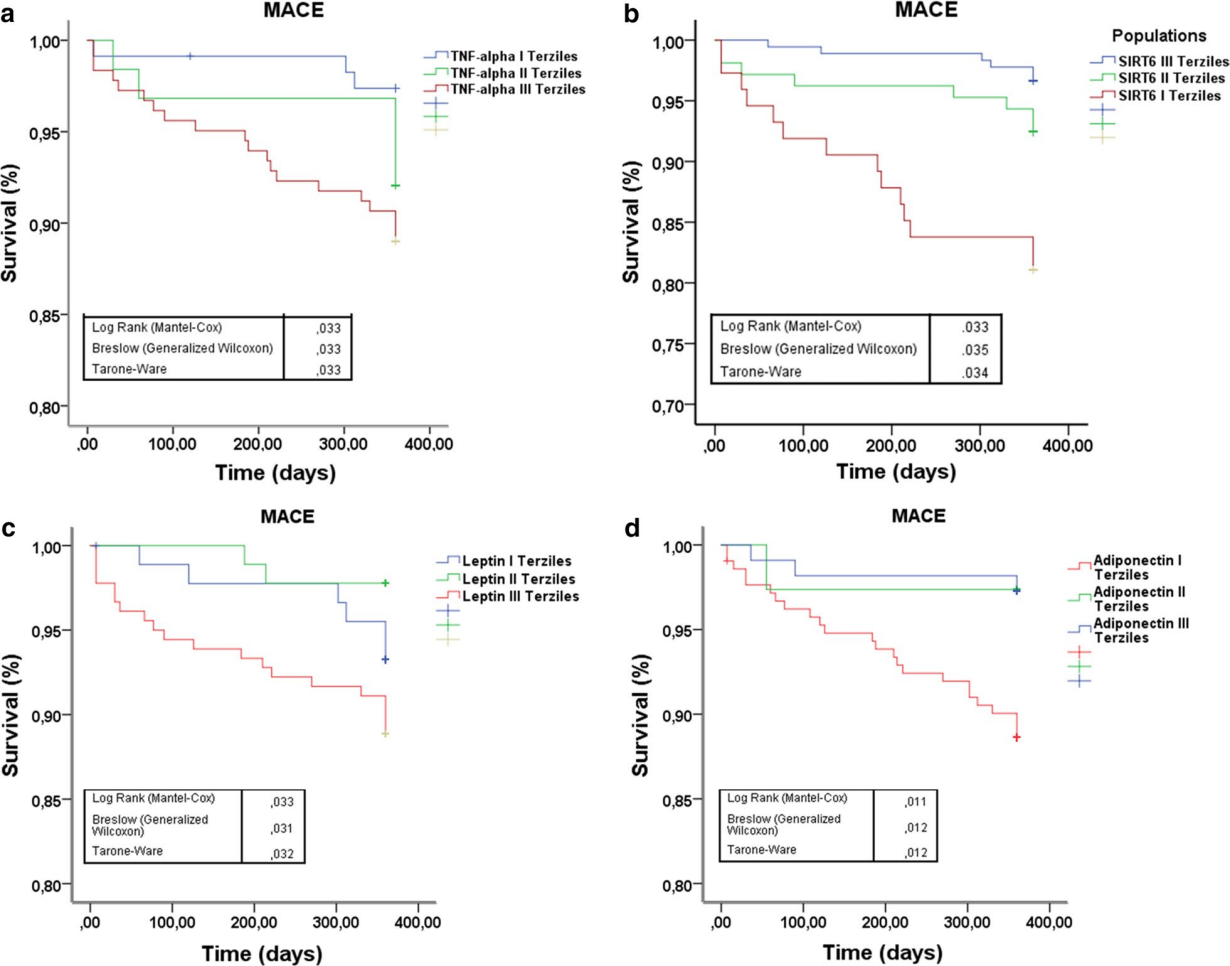
Kaplan–Meier survival curves according to TNF-α (**a**), SIRT6 (**b**) and leptin (**c**) and adiponectin (**d**) terziles. SPSS version 23.0 (IBM statistics) was used for all statistical analyses. Overall survival and event-free survival are presented using, and compared using the log-rank test

360 NG-patients and 266 prediabetic patients meet inclusion criteria. Among prediabetics, 50 patients (19%) had fasting glucose criterion, 26 patients (10%) had post-prandial glucose criterion, 130 patients (49%) had HbA1c criterion and the remaining 60 patients (22%) patients had two or more criteria for prediabetes diagnosis. All our prediabetic patients meet ADA criteria [18] for treatment with metformin (Table 1). Nevertheless, only 89 prediabetic patients were current-metformin-users (mean metformin dosage = 592.2 ± 167.7 mg/die), and 177 were never-metformin-users. During 1-year follow-up, 13 prediabetic patients (10 never-metformin-users and 3 current-metformin-users) developed type 2 diabetes and 26 (7 never-metformin-users and 19 current-metformin-users) reverted to normoglycemia, and were excluded from study (Fig. 1). After propensity score matching (PSM) for anthropometric characteristics and cardiovascular risk factors total of 180 NG-patients were matched to 180 prediabetic patients, (Table 1). After PSM, 58 current-metformin-users were matched to 58 never-metformin-users (Table 1), there were no differences between the criteria for the diagnosis of prediabetes. The mean (± SD) duration of metformin treatment was 21 ± 5.7 months. Prediabetic patients showed higher leptin to adiponectin ratio and TNF-α levels, whereas levels of SIRT6 were lower in pericoronary fat specimens compared to NG patients (Figs. 2, 3, 4) (P < 0.001). In addition, there were no significant differences in inflammatory tone, leptin to adiponectin ratio and MACE among pre-diabetic patients identified with the different diagnostic criteria (data not shown). In pericoronary fat specimens from prediabetic never-metformin-users, leptin to adiponectin ratio and TNF-α levels were significantly higher compared to current-metformin-users (P < 0.001) (Figs. 2, 3). Noticeable, SIRT6 level in pericoronary fat specimens from prediabetic never-metformin-users was consistently lower compared to current-metformin-users (P < 0.001) (Fig. 4). Remarkably, regression analysis evidenced a relationship between pericoronary fat leptin to adiponectin ratio and TNF-α levels in the overall study population. The analysis showed that values of pericoronary TNF-α content (dependent variables) increased when fat pericoronary leptin to adiponectin ratio (independent variable) increased, while the other independent variables are held fixed (Fig. 5a). In addition, regression analysis evidences a relationship between pericoronary fat leptin to adiponectin ratio and SIRT6 levels in the overall study population. The analysis showed that values of pericoronary SIRT6 content (dependent variables) decreased when fat pericoronary leptin to adiponectin ratio (independent variable) increased, while the other independent variables were held fixed (Fig. 5b). The MACE was 9.4% (n = 17) (cardiovascular death N = 3, 1.6%; HF N = 6, 3.3%; re-infarction N = 8, 4.4%) in all prediabetics and 3.3% (N = 6) (cardiovascular death N = 1, 0.5%; HF N = 2, 1.1%; re-infarction N = 3, 1.7%) in NG-patients (Fig. 6a). Prediabetic current-metformin-users presented a significantly lower rate of MACE compared to prediabetic never-metformin-users (Fig. 6b). The MACE was 20.7% (n = 12) (cardiovascular death N = 2, 3.4%; HF N = 4, 6.9%; re-infarction N = 6, 10.3%) in never-metformin-users and 6.9% (N = 4) (cardiovascular death N = 1, 1.7%; HF N = 1, 1.7%; re-infarction N = 2, 3.4%) in NG-patients. The multivariable logistic regression, model predicting MACE was adjusted for age, sex, hypertension, dyslipidemia, smoking history, family history, baseline no-diabetic therapies, metabolic parameters, and coronary lesions. Moreover, in order to translate the pericoronary fat inflammatory tone *status* into real clinical endpoints, the MACE stratified by leptin to adiponectin ratio, TNF-α and SIRT6 levels was further evaluated. As shown in Fig. 7, patients with lower leptin to adiponectin ratio, lower TNF-α levels, and higher SIRT6 had a lower number of events.

**Table 1:** Baseline clinical characteristics of patients with AMI matched by propensity score analysis

	Normoglycemic patients	Prediabetic patients	P	Prediabetic never metformin users	Prediabetic current metformin users	P
N	180	180		58	58	
Mean age (years)	68.2 ± 6.8	67.1 ± 6.3	0.082	66.2 ± 5.4	67.1 ± 4.9	0.345
Sex (M/F)	99/81	96/84	–	33/25	34/24	–
BMI (kg/m^2^)	27.1 ± 1.6	27.4 ± 1.9	0.088	27.3 ± 0.7	26.9 ± 1.2	0.140
Systolic blood pressure (mmHg)	130.8 ± 13.1	132.9 ± 10.9	0.064	131.2 ± 8.4	133.1 ± 7.7	0.945
Diastolic blood pressure (mmHg)	79.2 ± 6.5	79.3 ± 6.6	0.898	77.7 ± 6.4	79.4 ± 6.7	0.154
Heart rate (bpm)	87.2 ± 8.1	86.9 ± 8.6	0.744	85.9 ± 5.6	87.2 ± 8.8	0.357
Prediabetes diagnosis criteria						
Fasting plasma glucose, n (%)	–	39 (21.6)	–	10 (17.2)	11 (18.9)	0.488
Post-prandial glucose, n (%)	–	18 (10)	–	11 (18.9)	10 (17.2)	0.502
HbA1c, n (%)	–	102 (56.7)	–	24 (41.4)	25 (43.1)	0.493
Two or more criteria, n (%)	–	21 (11.6)	–	13 (22.4)	12 (20.7)	0.559
Risk factors						
Hypertension, n (%)	143 (79.4)	144 (80.1)	0.500	49 (84.5)	51 (87.9)	0.394
Hyperlipemia, n (%)	112 (62.2)	110 (61.1)	0.457	49 (84.5)	47 (81.1)	0.403
Cigarette smoking, n (%)	141 (78.3)	142 (78.9)	0.500	47 (81.1)	51 (87.9)	0.221
Active treatments						
β-blockers, n (%)	104 (57.8)	106 (58.9)	0.457	43 (74.1)	41 (70.7)	0.418
ACE inhibitors, n (%)	76 (42.2)	74 (41.1)	0.457	22 (37.9)	27 (46.6)	0.226
Angiotensin receptor blockers, n (%)	88 (48.9)	75 (41.7)	0.102	25 (43.1)	22 (37.9)	0.353
Calcium inhibitor, n (%)	100 (55.6)	105 (58.3)	0.335	37 (63.8)	41 (70.7)	0.277
Nitrate, n (%)	46 (25.6)	34 (18.9)	0.081	7 (12.1)	9 (15.5)	0.394
Statins, n (%)	92 (51.1)	100 (55.6)	0.230	32 (55.2)	28 (48.3)	0.289
Thiazide diuretic, n (%)	55 (30.6)	56 (31.1)	0.500	22 (37.9)	25 (43.9)	0.353
Aspirin, n (%)	115 (63.9)	118 (65.6)	0.413	37 (63.8)	38 (65.5)	0.500
Thienopyridine, n (%)	24 (13.3)	24 (13.3)	0.562	6 (10.3)	7 (12.1)	0.500
Laboratory analyses						
Fasting plasma glucose (mg/dl)	86.5 ± 6.5	110 ± 7.3	0.001	111.5 ± 9.3	110.9 ± 7.6	0.762
Post-prandial glucose (mg/dl)	106 ± 24	132 ± 36	0.001	131 ± 33	132 ± 29	0.658
HbA1c (%)	5.1 ± 0.3	6.2 ± 03	0.001	6.1 ± 0.3	6.0 ± 0.4	0.463
Cholesterol (mg/dl)	205.9 ± 21.1	205.1 ± 22.1	0.670	203.2 ± 19.1	206.6 ± 19.5	0.353
LDL-cholesterol (mg/dl)	127.4 ± 20.9	131.3 ± 21.5	0.080	128.6 ± 29.4	133.1 ± 18.9	0.198
HDL-cholesterol (mg/dl)	38.3 ± 3.5	38.1 ± 3.5	0.764	37.8 ± 3.3	37.5 ± 3.5	0.587
Triglycerides (mg/dl)	181.5 ± 19.6	182.6 ± 22.1	0.621	185.1 ± 23.6	179.6 ± 17.3	0.154
Creatinine (mg/dl)	0.99 ± 0.13	0.98 ± 0.19	0.399	0.98 ± 0.17	0.97 ± 0.16	0.833
hs-cTnT (ng/l)	147.6 ± 32.7	149.6 ± 25.9	0.411	149.9 ± 32.1	149.2 ± 26.6	0.604
Leptin (pg/ml)	27.9 ± 12.9	93.9 ± 28.1	0.001	111.1 ± 20.1	81.6 ± 24.8	0.001
Adiponectin (pg/ml)	116.1 ± 22.5	58.1 ± 23.3	0.001	45.8 ± 19.3	70.9 ± 18.7	0.001

Data are mean ± SD or n (%)

## Discussion

Here, we show that pericoronary fat from prediabetic patients is characterized by an increase of leptin and a reduction of adiponectin contents, as evidenced by an increase in the leptin to adiponectin ratio, and by a pro-inflammatory tone, as indicated by altered levels of SIRT6 and TNF-α. Moreover, as a consequence of these changes, MACE after AMI were greater in prediabetic patients than normoglycemics patients. In fact, patients with higher pericoronary fat inflammatory tone and leptin to adiponectin ratio had higher number of events. Of note, metformin therapy in prediabetic patients blunted these alterations, as current-metformin-users showed lower pro-inflammatory tone levels, leptin-to adiponectin ratio pericoronary fat and lower MACE compared to never-metformin-users. Finally, to the best of our knowledge, this is the first study showing that pericoronary fat pro-inflammatory tone and adipokine changes during AMI are amplified and might be responsible for poor outcomes in prediabetic patients. In this framework, modulation of SIRT6, TNF-α, leptin and adiponectin levels in pericoronary fat might have an important role to improve the poor outcome of prediabetic patients with AMI.

Previous studies showed an increased inflammation in pericoronary fat around culprit lesions compared to nonculprit lesions and around coronary spasm in patients with AMI [8, 21]. Moreover, perivascular fat depots may exert a protective modulation of vascular function and energy partition in a healthy situation, but their expansion turns them into an adverse lipotoxic, prothrombotic, and proinflammatory organ, as described in type 2 diabetic patients [22]. However, these studies did not provide any evidence about pericoronary fat inflammatory tone and adipokine changes in subgroups of high-risk patients, such as those found in prediabetic patients, or assess the specific pathway transducing environmental stimuli in inflammatory tone overexpression. In our study, TNF-α and leptin contents were more abundant in prediabetic patients along with a less abundant content of anti-inflammatory molecules, such as adiponectin and SIRT6, suggesting the presence of an active inflammatory reaction in prediabetic coronary fat. In agreement with the difference in inflammatory tone and leptin-to adiponectin ratio pattern, the pericoronary fat appears different with regard to inflammation, but not in the degree of vessel stenosis, suggesting that prediabetic and normoglycemic fat are only different in regard to inflammatory burden. These data are consistent with previous findings that certain adipokines, pro-inflammatory cytokines, and chemokines regulate adipose tissue function and metabolic processes that contribute to cardiovascular risk in patients with type 2 diabetes and obesity [23]. As background for this association, for the first time, we observed an association between inflammatory tone and leptin-to adiponectin ratio in pericoronary fat and the 12-months outcomes in prediabetic patients with AMI. Although it is well recognized that inflammation is responsible for post-AMI outcomes in patients with metabolic disorders [24], the mechanism by which prediabetic milieu may be involved in inflammatory process of prediabetic coronary fat is not fully clarified. In this context, our data suggest a novel mechanism by which prediabetic milieu, increasing leptin levels, may mediate inflammatory activity in prediabetic pericoronary fat. Of note, it has been shown high leptin levels along less adiponectin levels in prediabetic in subcutaneous adipose tissue [25]. Remarkably, altered secretion of leptin and adiponectin accelerate a chronic, proinflammatory profile with, thereby exacerbating cardiometabolic disease [26]. Moreover, adipokines regulate intracellular signaling pathways involving SIRT1 in mice diabetic hearts [27]. Thus, we can speculate that increased leptin content in coronary fat, as a consequence of prediabetic milieu, may enhance the inflammatory tone reducing SIRT6, possibly representing a crucial step in the pathophysiology of poor outcomes seen in prediabetic patients after AMI.

The present findings also show a protective effect of metformin therapies on cardiovascular outcomes in prediabetic patients after AMI. Indeed, diabetic patients treated with metformin had lowest incidence of cardiovascular events [28]. Moreover, in failing hearts, metformin improves myocardial energy metabolic status via several mechanisms, including activation of AMP-activated protein kinase (AMPK), regulation of lipid and glucose metabolism and increased nitric oxide (NO) bioavailability [29] and in T2DM patients metformin use is independently associated with a lower below-the-knee arterial calcification score [30]. Indeed, at the same level of blood glucose levels, prediabetic patients receiving metformin had the lowest level of inflammatory tone and leptin-to adiponectin ratio in pericoronary fat. Thus, patients receiving metformin had lesser inflammatory processes in pericoronary fat contributing to cardiovascular risk than prediabetic patients without metformin therapy. In particular, the reduced leptin content seen in pericoronary fat of the patients receiving metformin suggests decreased SIRT6 inactivation and hence the increase of inflammatory tone. Interestingly antidiabetic drugs such as sitagliptin, metformin, pioglitazone, liraglutide and empagliflozin were shown to reduce leptin levels in diabetic patients [31]. Moreover, metformin has a direct protective role to ameliorate the proinflammatory response through sirtuin-induction inhibiting p65-acetylation and thus reducing NF-kB activation in peripheral blood monocytes of patients with carotid artery atherosclerosis [32]. Activation of SIRTs appears to have beneficial effects on inflammatory tone [33]. In particular, the increased expression of SIRT 6 protects from fatty infiltration and inflammation [34]. Thus, the availability and safety of SIRT6 activators such as metformin provide an excellent opportunity to modulate inflammatory tone in human pericoronary fat. Accordingly, the current-metformin-users presented lowest levels of pericoronary fat pro-inflammatory tone, as SIRT6 levels were increased and TNF-α levels were reduced.

### Study limitations

Some limitations of our study need consideration. First, because it was an observational study, the results did not imply causality. Second, the study had a small number of patients. In addition, since most of the subjects had medical treatment, such as statins and antihypertensive drugs, our data on risk factors, including the lipid profile, may reflect the effects of medications to some extent. Third, we performed an observational study without any randomization. Therefore, further detailed analyses in a large number of randomized patients are required to clarify our findings.

## Conclusions

These data may have important clinical implication. Indeed, metformin therapy in pre-diabetic patients may be useful to reduce the progression of type 2 diabetes and to prevent cardiovascular outcomes before diabetes development. Moreover, these novel evidences may be useful to reconsider metformin prescription in high risk population, as prediabetic patients, for which metformin is prescribed only for 3.7% despite ADA recommendation [35].

## Data Availability

All data and material are available.

## Abbreviations

AMI: acute myocardial infarction
CABG: coronary artery bypass grafting
eGFR: estimated glomerular filtration rate
IL-6: interleukin-6
MACE: Major-Adverse-Cardiac-Events
NG: normoglycemic patients
NYHA: New York Heart Association
SIRT6: sirtuin-6
TNF-α: tumor necrosis factor-α
